# Evaluating the Efficacy of a Digital Therapeutic Intervention for Temporomandibular Disorders: Multicenter, Randomized, Sham-Controlled Trial

**DOI:** 10.2196/83545

**Published:** 2025-10-24

**Authors:** Sang-Yoon Park, Sung-Woon On, Sangmin Yi, Sung-Ah Che, Bongju Kim, Soo-Hwan Byun, Byoung-Eun Yang

**Affiliations:** 1 Department of Oral and Maxillofacial Surgery Hallym University Sacred Heart Hospital Anyang Republic of Korea; 2 Division of Oral and Maxillofacial Surgery Hallym University Dongtan Sacred Heart Hospital Hwaseong Republic of Korea; 3 Dental Life Science Research Institute Seoul National University Dental Hospital Seoul Republic of Korea

**Keywords:** temporomandibular joint disorders, digital health, digital therapeutics, mobile apps, cognitive behavioral therapy, randomized controlled trial

## Abstract

**Background:**

Temporomandibular disorders (TMDs) are common chronic musculoskeletal conditions associated with pain, jaw dysfunction, and impaired quality of life and influenced by behavioral and psychological factors. Digital therapeutics (DTx) may improve access to standardized behavioral interventions, but their clinical efficacy for TMD has not been adequately established.

**Objective:**

This study aimed to investigate the efficacy of a mobile-based DTx intervention (Clickless DTx TMD-01) compared with a sham app in reducing pain and improving functional and psychological outcomes in patients with TMD.

**Methods:**

We conducted a multicenter, double-blind, randomized, sham-controlled trial at 2 university hospitals in South Korea. Adults aged between 19 and 65 years with a diagnosis of TMD according to the diagnostic criteria for TMD were randomly assigned (1:1) to the intervention group or sham control. The intervention was delivered via the DTx mobile app and included TMD-related disease education, guided jaw exercises, behavioral habit tracking, psychoeducational content, guided meditation, and data-driven feedback. The sham app provided only symptom recording without therapeutic content. Participants were instructed to use the assigned app daily for 6 weeks. The primary outcome was change in pain intensity measured by a 100-mm visual analog scale. Secondary outcomes included maximum mouth opening and scores on the Jaw Functional Limitation Scale-20, Oral Behaviors Checklist, and Patient Health Questionnaire-4.

**Results:**

A total of 102 participants were randomly assigned (DTx group: n=50, 49%; sham group: n=52, 51%). For the per-protocol analysis, 44 (88%) of the 50 participants in the DTx group and 49 (94%) of the 52 participants in the sham group were included. At 6 weeks, the DTx group showed a significantly greater reduction in pain on the visual analog scale compared with the sham group (–33.64 vs –9.86; between-group difference –23.78, 95% CI –34.15 to –13.41; *P*<.001). Secondary outcomes also favored the DTx group, with improvements in maximum mouth opening (between-group difference 4.36 mm, 95% CI 1.92-6.81 mm; *P*<.001), Jaw Functional Limitation Scale-20 scores (–1.02, 95% CI –1.63 to –0.42; *P*=.005), and Oral Behaviors Checklist scores (–5.84, 95% CI –10.18 to –1.50; *P*=.009). No significant between-group difference was observed in Patient Health Questionnaire-4 scores. Adherence was acceptable, as all participants included in the final analysis met the use threshold of at least 66.7%. No serious adverse events were reported.

**Conclusions:**

This randomized, sham-controlled trial demonstrated that a mobile-based DTx intervention led to significant improvement in pain at 6 weeks compared with sham control, with additional benefits in jaw function and oral behaviors, although no significant between-group difference was observed in psychological distress. These findings suggest that DTx interventions may serve as a promising and scalable adjunct to conventional care for patients with TMD.

**Trial Registration:**

Clinical Research Information Service KCT0009493; https://tinyurl.com/567fexbj

## Introduction

Temporomandibular disorder (TMD) is a collective term for a range of clinical conditions affecting the masticatory muscles, temporomandibular joint, and associated structures, which can significantly impair daily functioning and quality of life owing to symptoms such as jaw pain, restricted movement, and joint sounds [1]. Globally, TMD affects up to 15% of the adult population and is more prevalent in women of reproductive age [2,3]. Recent trends suggest a rise in TMD diagnoses, potentially due to heightened stress, an increase in behavioral risk factors, and improved clinical awareness [3,4]. Etiologically, TMD involves a complex interplay of musculoskeletal, behavioral, and psychosocial factors [5,6].

Although conservative treatments for TMD—such as medication, occlusal splints, and physiotherapy—are commonly used, long-term patient management remains suboptimal [7]. Behavioral and psychological interventions have demonstrated efficacy but remain underused owing to limited accessibility and integration within dental practice settings [8,9]. A previous study highlighted that the integration of eHealth solutions into TMD care enhances health care provider–patient interaction, supports patient self-management, and strengthens multidisciplinary collaboration [10]. Given the chronic and behaviorally influenced nature of TMD, it is suitable for digital therapeutic (DTx) interventions [11]. Digital platforms can facilitate structured education, guided exercises, stress management, and real-time symptom tracking, aligning with personalized care models [12].

DTx, which are evidence-based, software-driven medical interventions, aim to treat or manage diseases by modifying patient behavior and supporting clinical outcomes [13]. These regulated interventions are often prescribed as complements or alternatives to standard care. As health care systems face increasing burdens from chronic diseases and growing demand for patient-centered solutions, DTx interventions represent a robust solution to enhance adherence and reduce dependency on in-person care [14].

Notably, DTx, when used as an adjunctive therapy, may provide greater symptom relief than conventional therapy alone by targeting behavioral factors often overlooked in standard TMD care [11]. A previous pilot trial provided preliminary evidence of the feasibility and clinical utility of a smartphone-based DTx app for TMD management, featuring interactive modules and self-guided content that improved pain levels and jaw mobility [11]. Building on these findings, this study evaluated a refined version of the DTx intervention that features extended treatment duration, enhanced interactivity, and adherence-monitoring capabilities through a rigorously designed multicenter, double-blind, randomized, sham-controlled trial. We hypothesized that a DTx-based intervention would yield superior therapeutic benefits compared with sham control. By assessing its efficacy and safety over an extended intervention period, we aimed to generate meaningful evidence supporting the adoption of DTx in the management of TMD and further establish DTx as a viable tool in orofacial pain treatment paradigms.

## Methods

### Study Design

This multicenter, prospective, double-blind, randomized, sham-controlled, superiority trial was designed to evaluate the efficacy and safety of a DTx intervention in patients diagnosed with TMD in combination with conventional treatment. The trial compared clinical outcomes between a group using the DTx smartphone app and a control group using a sham app over a 6-week period, with assessments across 4 scheduled visits.

### Ethical Considerations

The trial was conducted in accordance with the Declaration of Helsinki and Good Clinical Practice guidelines and followed the CONSORT (Consolidated Standards of Reporting Trials) 2025 guidelines. This trial was reviewed and approved by the institutional review boards of both participating centers (Hallym IRB 2024-04-001-006 and HDT IRB 2024-04-001-004) as well as by the South Korean Ministry of Food and Drug Safety (1700). The study was registered on the Clinical Research Information Service (KCT0009493; registration date April 30, 2024).

All participants provided written informed consent before enrollment. Participant data were pseudonymized using study ID numbers and stored in a secure, password-protected electronic data capture system. Only authorized study investigators had access to the dataset, and no personally identifiable information was included in the analysis.

Participants received financial compensation (including transportation expenses) of approximately ₩500,000 (approximately US $350) upon completion of the trial. No deviations from the registered protocol occurred during the study.

### Sample Size Calculation

The clinical evaluation period was set to 6 weeks based on prior studies, which generally assessed TMD outcomes over shorter durations [15]. Supporting evidence from trials involving cognitive behavioral therapy showed significant reductions in pain scores on a visual analog scale (VAS) over a period of 5.9 to 6 weeks [16]. Assuming a mean VAS improvement of 3.26 mm in the intervention group and 2.06 mm in the sham group, with an SD of 1.579 mm, a 2-tailed α of .05, and 90% power, the required sample size was 38 participants per group (calculated using G*Power [version 3.1, Heinrich Heine University]). Allowing for a 25% dropout rate, the final enrollment target was 102 participants (n=51, 50% per group).

### Participant Selection

Participants were recruited from outpatient clinics at the departments of oral and maxillofacial surgery at 2 university hospitals in South Korea (Hallym University Sacred Heart Hospital and Hallym University Dongtan Sacred Heart Hospital). Both institutions serve large regional populations and maintain specialized oral and maxillofacial surgery units that provide comprehensive diagnostic and therapeutic services for TMDs. Recruitment was conducted through direct referral during clinical visits, as well as posters and flyers displayed at the clinics.

Participants were eligible if they were adults aged between 19 and 65 years, had a diagnosis of TMD based on the diagnostic criteria for TMDs (DC/TMD) [17], had available clinical and radiographic findings, and provided written informed consent. Participants were excluded if they had structural joint diseases (eg, ankylosis or tumors), traumatic injuries (eg, mandibular fracture), neuromuscular or severe metabolic disorders, neuropsychiatric illnesses, or recent use of sleep medications or illicit substances; were undergoing orthodontic treatment; were pregnant or planning pregnancy; or had participated in another clinical trial within the prior 4 weeks.

### Randomization and Blinding

Participants were randomly assigned (1:1) to the DTx or sham app group using stratified block randomization by site, implemented through the PLAN procedure in SAS (version 9.4 or higher; SAS Institute Inc). An independent randomization statistician created and safeguarded the allocation list, which was integrated into an interactive web-based response system for automated assignment upon eligibility confirmation.

To maintain blinding, the randomization process was separated from outcome assessment and data analysis. The sham app mimicked the appearance and functionality of the DTx app but lacked therapeutic content. Both participants and investigators were blinded to grouping. Nonblinded clinical research coordinators monitored adherence and adverse events without engaging in outcome assessments. Randomization codes were disclosed only after database freezing to secure data integrity.

### Intervention Procedures

#### DTx Group

The intervention group used the Clickless DTx app (version 2.0), a prescription DTx designed for patients with TMD. Over a 6-week period, the program delivered combined core components, including concise TMD-related disease education, guided jaw exercises (Rocabado 6×6×6 protocol), behavioral habit tracking, psychoeducational content, guided meditation, and data-driven feedback. Upon first log-in, participants completed an initial onboarding questionnaire regarding symptoms and behavioral patterns, allowing the app to generate a personalized daily checklist aimed at promoting consistent engagement and individualized behavioral change. The app featured both video-guided and facial recognition–based exercise modes. Users were instructed to engage with the app for 5 to 10 minutes daily. A “Jaw health diary” component enabled self-monitoring of pain, stress, sleep quality, splint use, and relevant behavioral triggers. Visual feedback via weekly reports helped reinforce adherence and highlight symptom trends. Participants with less than 66.7% use compliance were excluded from the analysis. Supportive check-in calls were provided during weeks 1, 3, and 5 to enhance user engagement and address technical barriers.

#### Sham Group

The sham app was visually identical but lacked all therapeutic content, enabling only weekly data input for outcome measurement through passive user participation, without any interactive or therapeutic functions. It served as a placebo control to isolate the effect of the active intervention.

**Figure 1:**
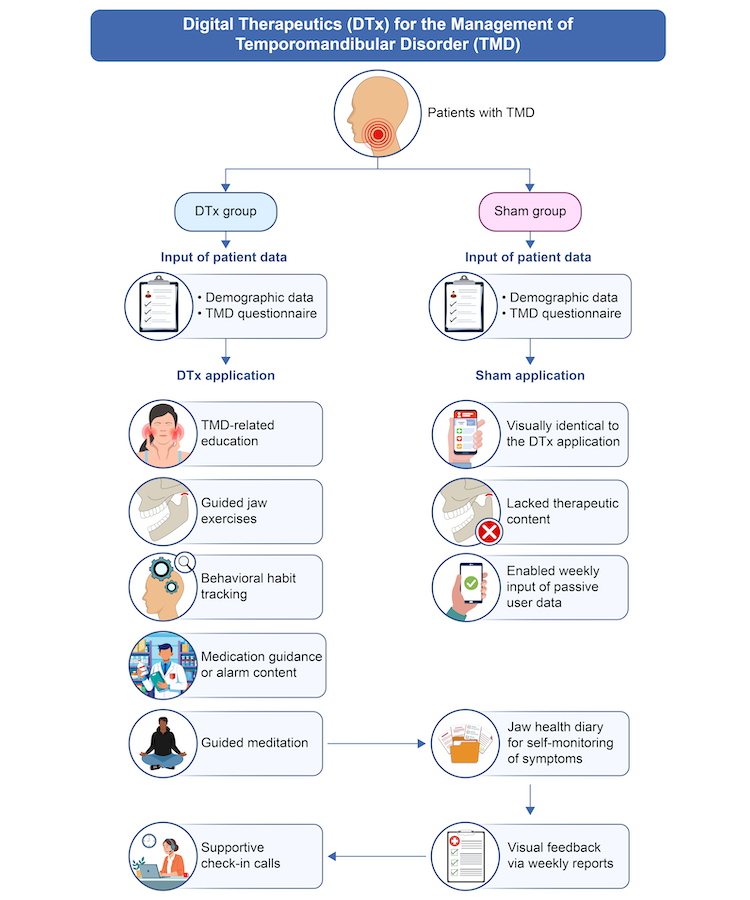
Intervention structure: DTx versus sham app.

All participants in both groups continued to receive treatment as usual (TAU) during the study period, ensuring that no participant was deprived of standard clinical care. The app structure is detailed in Figure 1.

### Outcome Measures

#### Primary End Point

The primary outcome was the change in pain intensity, assessed using a 100-mm horizontal VAS. Participants rated their perceived pain from 0 (no pain) to 100 (worst imaginable pain) at baseline and at weeks 2, 4, and 6 (endline).

#### Secondary End Points

Secondary outcomes included maximal mouth opening (MMO, mm), measured as the interincisal distance using a plastic millimeter ruler. Measurements of MMO were conducted during outpatient visits by oral and maxillofacial surgery faculty members (BEY, SHB, SMY, and SWO) who provided clinical care to the participants. The Jaw Functional Limitation Scale-20 (JFLS-20), Oral Behavior Checklist (OBC), and Patient Health Questionnaire-4 (PHQ-4) were administered in accordance with the standardized DC/TMD Axis II assessment protocol [17].

Outcome measurements were conducted in person through paper-based questionnaires during scheduled clinic visits at baseline and at weeks 2, 4, and 6. In addition, app-based assessments were performed at weeks 1, 3, and 5 to capture interim symptom data, specifically limited to the OBC and PHQ-4. Adverse events, including both serious and nonserious events, were monitored at each follow-up visit and systematically recorded. Completed questionnaires were collected on site and subsequently entered into a secure electronic database by trained research staff who were not involved in patient care. To ensure data quality, all entries were checked through range validation and cross-verification procedures.

### Statistical Analysis

All data were securely stored and managed using a validated electronic data capture system with encrypted access. All statistical analyses were effectively conducted in a per-protocol population, defined as participants who completed the week 6 assessment and demonstrated at least 66.7% adherence to the intervention, as minimal engagement with the app would not constitute meaningful exposure to the therapeutic content. For all outcomes, normality was assessed before analysis. Within-group differences from baseline to week 6 were evaluated using paired 2-tailed *t* tests. Between-group differences in change scores were analyzed using independent *t* tests. When the assumption of normality was not met, nonparametric Wilcoxon rank sum tests were used. A 2-tailed *P* value <.05 was considered statistically significant. Missing data were minimal and limited to participants who did not complete the week 6 follow-up. These participants were excluded from the primary per-protocol analysis. An intention-to-treat (ITT) analysis was also conducted, with missing data handled by last-observation-carried-forward imputation. All statistical analyses were performed using SAS (version 9.4).

## Results

### Demographic Data and Study Flow

**Figure 2:**
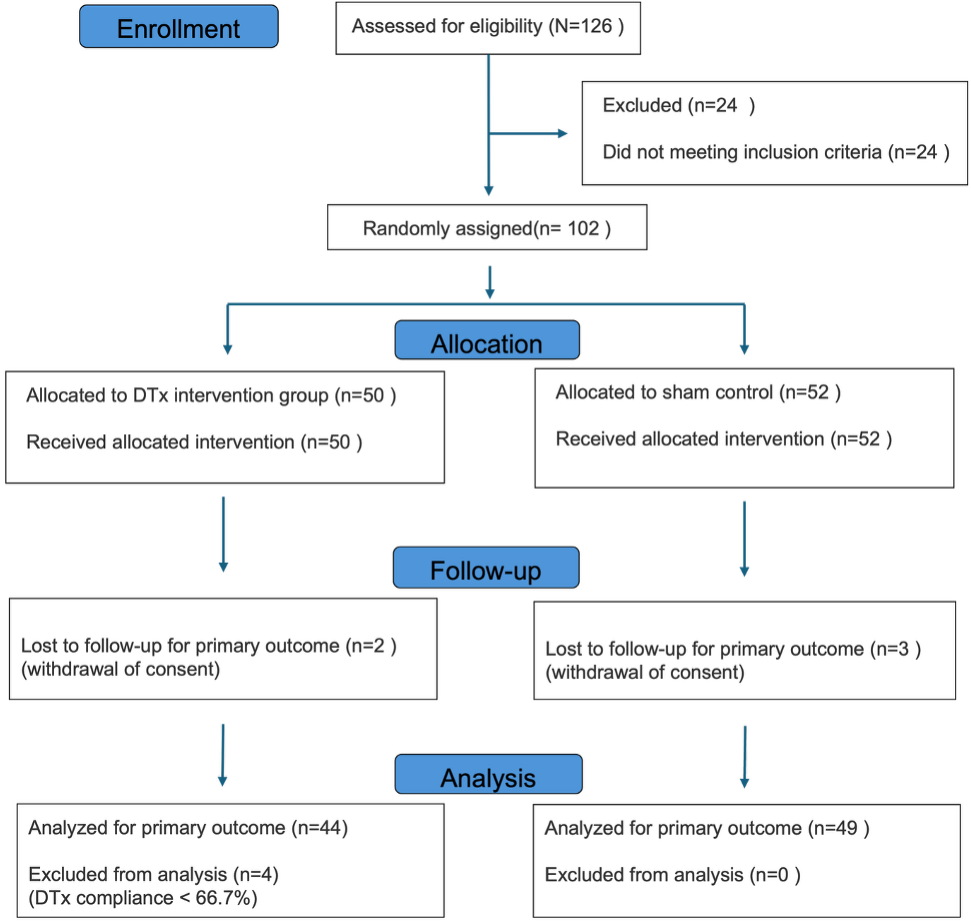
CONSORT flow diagram.

From June 2024 to June 2025, participants were recruited from 2 institutions. A total of 126 participants were assessed for eligibility, and 102 (81%) were subsequently enrolled and randomly assigned (Hallym University Sacred Heart Hospital—DTx group: n=34, 33%; sham group: n=33, 32%; Hallym University Dongtan Sacred Heart Hospital—DTx group: n=18, 18%; sham group: n=17, 17%). Of the 102 participants, 50 (49%) were assigned to the Clickless DTx group and 52 (51%) to the sham control group, with all participants receiving their allocated intervention. For the primary outcome analysis, we included 44 (88%) of the 50 participants in the DTx group (n=4, 8% were excluded because of protocol noncompliance [defined as intervention adherence below 66.7%], and n=2, 4% were lost to follow-up). In the sham control group, 3 (6%) of the 52 participants were lost to follow-up, leaving 49 (94%) participants for inclusion in the analysis. The participant flow through each stage of the trial is illustrated in Figure 2. No additional item-level missing data were observed among participants who completed the study assessments. Accordingly, the final analysis included 93 participants in the per-protocol population (DTx group: n=44, 47%; sham group: n=49, 53%). This approach ensured that the outcome comparisons reflected participants with meaningful exposure to the intervention. All participants completed the trial without experiencing any serious harm or adverse events. Demographic characteristics were comparable between groups. Full baseline data are summarized in Table 1. The characteristics of participants included in the per-protocol analysis are provided in Multimedia Appendix 1.

**Table 1:** Baseline demographic data.

Characteristics				Intervention (DTx^a^) group (n=50)		Sham control group (n=52)		*P* value
**Age (years)**								.09
	Mean (SD)			37.26 (11.46)		41.25 (12.27)		
**Sex, n (%)**								.61^b^
	Male			14 (28)		17 (33)		
	Female			36 (72)		35 (67)		
**Height (cm)**								.38
	Mean (SD)			164.76 (8.05)		166.27 (9.27)		
**Weight (kg)**								.16
	Mean (SD)			62.31 (12.07)		66.25 (15.54)		
**Smoking status, n (%)**								.66^b^
	Never smoker			42 (84)		44 (85)		
	Former smoker			4 (8)		6 (12)		
	Current smoker			4 (8)		2 (4)		
**Cigarette consumption (per week)**								
	**Former smoker**							.17
		Mean (SD)	28.00 (29.47)		65.33 (42.74)			
	**Current smoker**							.77
		Mean (SD)	46.00 (33.48)		57.50 (60.10)			
**Alcohol consumption status, n (%)**								.58^b^
	Never drinker			19 (38)		21 (40)		
	Former drinker			6 (12)		3 (6)		
	Current drinker			25 (50)		28 (54)		
**Alcohol consumption (per month)**								
	**Former drinker**							.09
		Mean (SD)	6.50 (7.04)		18.67 (12.22)			
	**Current drinker**							.14
		Mean (SD)	13.64 (19.90)		7.29 (7.93)			

^a^DTx: digital therapeutic.

^b^*P* values were calculated using the chi-square test or the Fisher exact test, as appropriate; all other *P* values were obtained using a 2-sample *t* test.

### Primary and Secondary Outcomes

**Figure 3:**
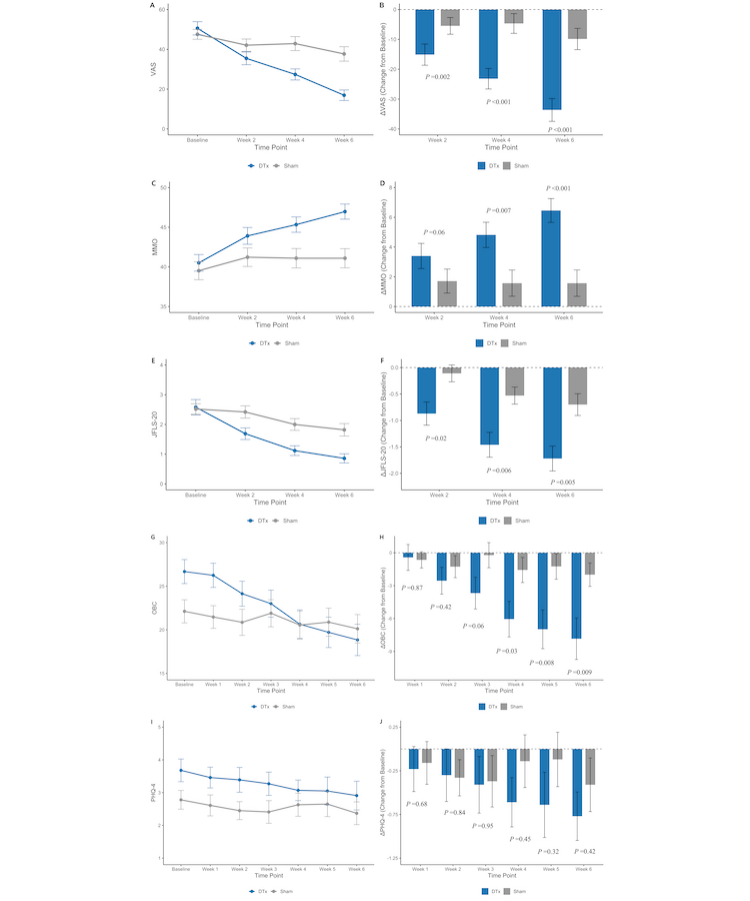
Primary and secondary outcomes. JFLS-20: Jaw Functional Limitation Scale-20; MMO: maximum mouth opening; OBC: Oral Behavior Checklist; PHQ-4: Patient Health Questionnaire-4; VAS: visual analog scale. Higher-resolution version of this figure is available in Multimedia Appendix 2.

The results for the 5 outcome variables are presented in Tables 2 and 3 and illustrated in Figure 3.

**Table 2:** Comparison of outcomes between digital therapeutic (DTx) and sham groups over time (per-protocol analysis; n=93).

		DTx (n=44), mean (SD)	Sham (n=49), mean (SD)	Mean difference (95% CI)	*P* value
**VAS^a^**					
	Baseline	50.57 (22.13)	47.55 (17.41)	3.02 (−5.14 to 11.18)	.36^b^
	Wk 2	35.46 (21.15)	42.08 (21.62)	−6.63 (−15.46 to 2.20)	.16^b^
	Wk 4	27.39 (18.47)	42.88 (24.50)	−15.49 (−24.51 to −6.47)	.*003*^b,c^
	Wk 6	16.93 (17.46)	37.69 (25.38)	−20.76 (−29.67 to −11.86)	*<.001^b^*
**MMO^d^**					
	Baseline	40.51 (6.94)	39.52 (7.94)	0.99 (−2.10 to 4.08)	.53
	Wk 2	43.91 (7.01)	41.22 (8.16)	2.68 (−0.47 to 5.83)	.09
	Wk 4	45.33 (6.41)	41.09 (8.58)	4.24 (1.09 to 7.38)	.*009*
	Wk 6	46.97 (6.30)	41.09 (8.44)	5.87 (2.78 to 8.97)	*<.001*
**JFLS-20^e^**					
	Baseline	2.58 (1.69)	2.52 (1.24)	0.06 (−0.56 to 0.67)	.74^b^
	Wk 2	1.69 (1.28)	2.42 (1.44)	−0.73 (−1.30 to −0.16)	.*02*^b^
	Wk 4	1.12 (1.07)	2.00 (1.38)	−0.88 (−1.39 to −0.36)	.*001*^b^
	Wk 6	0.86 (1.01)	1.82 (1.47)	−0.97 (−1.48 to −0.45)	*<.001^b^*
**OBC^f^**					
	Baseline	26.68 (9.13)	22.12 (9.27)	4.56 (0.76 to 8.36)	.*01*
	Wk 1	26.25 (9.19)	21.47 (9.11)	4.78 (1.01 to 8.55)	.*01*
	Wk 2	24.14 (9.50)	20.86 (10.51)	3.28 (−0.86 to 7.42)	.12
	Wk 3	23.00 (10.29)	21.90 (10.88)	1.10 (−3.27 to 5.48)	.62
	Wk 4	20.64 (10.87)	20.55 (11.16)	0.09 (−4.46 to 4.63)	.89^b^
	Wk 5	19.71 (11.58)	20.88 (11.23)	−1.17 (−5.87 to 3.53)	.76^b^
	Wk 6	18.84 (11.95)	20.12 (11.41)	−1.28 (−6.10 to 3.53)	.47^b^
**PHQ-4^g^**					
	Baseline	3.68 (2.30)	2.78 (2.00)	0.91 (0.02 to 1.79)	.*04*^b^
	Wk 1	3.46 (2.10)	2.61 (2.24)	0.84 (−0.06 to 1.74)	.06^b^
	Wk 2	3.39 (2.51)	2.45 (1.92)	0.94 (0.02 to 1.85)	.08^b^
	Wk 3	3.27 (2.36)	2.41 (2.42)	0.86 (−0.12 to 1.85)	.*04*^b^
	Wk 4	3.07 (2.08)	2.63 (2.46)	0.44 (−0.51 to 1.38)	.15^b^
	Wk 5	3.05 (2.82)	2.65 (2.67)	0.39 (−0.74 to 1.52)	.45^b^
	Wk 6	2.91 (2.44)	2.37 (2.43)	0.54 (−0.46 to 1.55)	.18^b^

^a^VAS: visual analog scale.

^b^*P* value was calculated using the Wilcoxon rank sum test due to nonnormal distribution; all other *P* values were obtained using an independent 2-sample *t* test.

^c^Italicization indicates values that met the significance threshold (*P*<.05).

^d^MMO: maximum mouth opening.

^e^JFLS-20: Jaw Functional Limitation Scale-20.

^f^OBC: Oral Behavior Checklist.

^g^PHQ-4: Patient Health Questionnaire-4.

**Table 3:** Comparison of changes from baseline between digital therapeutic (DTx) and sham groups over time (per-protocol analysis; n=93).

			ΔDTx (n=44)					ΔSham (n=49)				Mean difference (95% CI)			*P* value
			Mean (SD)		*P* value^a^		Mean (SD)			*P* value^a^					
**VAS^b^**															
	Wk 2	−15.11 (23.56)		*<.001^c^*		−5.47 (19.57)			.12		−9.64 (−18.53 to −0.75)		.*002*^d^		
	Wk 4	−23.18 (22.75)		*<.001*		−4.67 (23.11)			.16		−18.51 (−27.97 to −9.04)		*<.001^d^*		
	Wk 6	−33.64 (25.34)		*<.001*		−9.86 (24.96)			.008		−23.78 (−34.15 to −13.41)		*<.001*		
**MMO^e^**															
	Wk 2	3.40 (5.63)		*<.001*		1.70 (5.65)			.04		1.69 (−0.63 to 4.02)		.06^d^		
	Wk 4	4.82 (5.63)		*<.001*		1.57 (6.17)			.08		3.25 (0.80 to 5.69)		.*007*^d^		
	Wk 6	6.46 (5.31)		*<.001*		1.57 (6.20)			.08		4.36 (1.92 to 6.81)		*<.001^d^*		
**JFLS-20^f^**															
	Wk 2	−0.87 (1.44)		*<.001*		−0.11 (1.11)			.51		−0.77 (−1.30 to −0.24)		.*02*^d^		
	Wk 4	−1.46 (1.54)		*<.001*		−0.53 (1.13)			.002		−0.93 (−1.49 to −0.37)		.*006*^d^		
	Wk 6	−1.72 (1.54)		*<.001*		−0.70 (1.44)			.001		−1.02 (−1.63 to −0.41)		.*005*^d^		
**OBC^g^**															
	Wk 1	−0.43 (7.80)		.72		−0.65 (5.14)			.38		0.22 (−2.54 to 2.98)		.87		
	Wk 2	−2.55 (8.10)		*.04*		−1.27 (7.01)			.21		−1.28 (−4.39 to 1.83)		.42		
	Wk 3	−3.68 (9.58)		*.02*		−0.22 (8.01)			.85		−3.46 (−7.08 to 0.17)		.06		
	Wk 4	−6.05 (10.76)		*<.001*		−1.57 (8.01)			.18		−4.47 (−8.43 to −0.52)		*.03*		
	Wk 5	−6.98 (11.72)		*<.001*		−1.25 (8.14)			.29		−5.73 (−9.94 to −1.52)		*.008*		
	Wk 6	−7.84 (12.56)		*<.001*		−2.00 (7.48)			.07		−5.84 (−10.18 to −1.50)		*.009*		
**PHQ-4^h^**															
	Wk 1	−0.23 (1.71)		.54		−0.16 (1.71)			.50		−0.06 (−0.77 to 0.64)		.68^d^		
	Wk 2	−0.30 (2.00)		.33		−0.33 (1.45)			.14		0.03 (−0.70 to 0.76)		.84^d^		
	Wk 3	−0.41 (2.15)		.24		−0.37 (2.06)			.15		−0.04 (−0.91 to 0.83)		.95^d^		
	Wk 4	−0.61 (1.87)		.05		−0.14 (2.11)			.50		−0.47 (−1.30 to 0.35)		.45^d^		
	Wk 5	−0.64 (2.48)		.09		−0.12 (2.18)			.67		−0.51 (−1.47 to 0.44)		.32^d^		
	Wk 6	−0.77 (1.84)		*.007*		−0.41 (2.15)			.20		−0.36 (−1.19 to 0.46)		.42^d^		

^a^Statistically significant changes from baseline to each week were tested using a paired *t* test.

^b^VAS: visual analog scale.

^c^Italicization indicates values that met the significance threshold (*P*<.05).

^d^*P* value was calculated using the Wilcoxon rank sum test due to nonnormal distribution; all other *P* values were obtained using an independent 2-sample *t* test.

^e^MMO: maximum mouth opening.

^f^JFLS-20: Jaw Functional Limitation Scale-20.

^g^OBC: Oral Behavior Checklist.

^h^PHQ-4: Patient Health Questionnaire-4.

At baseline, the mean VAS scores were 50.57 (SD 22.13) in the DTx group and 47.55 (SD 17.41) in the sham group (*P*=.36). At 6 weeks, significant decreases in VAS scores were observed in both groups compared with baseline (DTx group: mean −33.64, SD 25.34; *P*<.001; sham group: mean −9.86, SD 24.96; *P*=.008). The mean VAS score at 6 weeks was significantly lower in the DTx group than in the sham group, with a between-group difference at 6 weeks of −20.76 (95% CI −29.67 to −11.86; *P*<.001). Additionally, the change in VAS scores from baseline to week 6 was significantly greater in the DTx group than in the sham group, with a between-group difference in change scores of −23.78 (95% CI −34.15 to −13.41; *P*<.001). These results are illustrated in Figures 3A and 3B.

For MMO, mean baseline measurements were 40.51 (SD 6.94) mm in the DTx group and 39.52 (SD 7.94) mm in the sham group (*P*=.53). At 6 weeks, mean MMO increased to 46.97 (SD 6.30) mm in the DTx group and 41.09 (SD 8.44) mm in the sham group. The increase was statistically significant only in the DTx group (*P*<.001). The mean MMO at 6 weeks was significantly greater in the DTx group than in the sham group, with a between-group difference of 5.87 mm (95% CI 2.78-8.97 mm; *P*<.001). The between-group difference in change from baseline to 6 weeks was 4.36 mm (95% CI 1.92-6.81 mm; *P*<.001). The MMO results are illustrated in Figures 3C and 3D.

The mean JFLS-20 global scores were 2.58 (SD 1.69) in the DTx group and 2.52 (SD 1.24) in the sham group (*P*=.74) at baseline. At 6 weeks, both groups showed significant reductions in mean scores: from baseline to 0.86 (SD 1.01) in the DTx group (within-group change: mean −1.72, SD 1.54; *P*<.001) and to 1.82 (SD 1.47) in the sham group (within-group change: mean −0.70, SD 1.44; *P*=.001). The reduction was significantly greater in the DTx group, with a between-group difference in change scores of −1.02 (95% CI −1.63 to −0.42; *P*=.005). The JFLS-20 global score results are illustrated in Figures 3E and 3F.

At baseline, the mean OBC scores were 26.68 (SD 9.13) in the DTx group and 22.12 (SD 9.27) in the sham group (*P*=.01). At 6 weeks, the mean scores decreased to 18.84 (SD 11.95) in the DTx group and 20.12 (SD 11.41) in the sham group; the decrease was statistically significant only in the DTx group (*P*<.001). The mean within-group change was −7.84 (SD 12.56) for the DTx group (*P*<.001) and −2.00 (SD 7.48) for the sham group (*P*=.07), indicating a greater reduction in the DTx group. The between-group difference in change scores was −5.84 (95% CI −10.18 to −1.50; *P*=.009). The OBC score results are illustrated in Figures 3G and 3H.

At baseline, the mean PHQ-4 scores were 3.68 (SD 2.30) in the DTx group and 2.78 (SD 2.00) in the sham group (*P*=.04). At 6 weeks, the mean scores decreased to 2.91 (SD 2.44) in the DTx group and 2.37 (SD 2.43) in the sham group; the reduction was statistically significant only in the DTx group (*P*=.007). The mean within-group change was −0.77 (SD 1.84) in the DTx group (*P*=.007) and −0.41 (SD 2.15) in the sham group (*P*=.20). At 6 weeks, the between-group difference in change scores was −0.36 (95% CI −0.57 to 1.11; *P*=.42). The PHQ-4 score results are illustrated in Figures 3I and 3J.

The results of the ITT analysis were comparable to those of the per-protocol analysis, with minor variations. Detailed results of the ITT analysis can be found in Multimedia Appendix 3.

## Discussion

### Principal Findings

This multicenter, double-blind, randomized, sham-controlled trial demonstrated that a DTx intervention, when combined with TAU, led to a significant reduction in pain at 6 weeks compared with sham. Additional benefits were observed in jaw mobility, perceived functional limitation, and oral behaviors, although no significant between-group difference was found in psychological distress. These findings suggest that a DTx may serve as a promising adjunctive intervention to conventional care for TMD, particularly for addressing both physical (axis I) and behavioral (axis II) components of the disorder.

### Comparison With Prior Work

Regarding pain intensity, the DTx group showed a significantly greater reduction in VAS at week 6 compared with the sham group. Moreover, the pain-relieving effect of the DTx appeared earlier and with greater magnitude compared with the sham group. These findings suggest that while conventional TAU alone may result in symptomatic relief over a 6-week period, the addition of a DTx leads to faster and more substantial clinical benefits. This pattern is consistent with previous randomized controlled trials involving DTx for chronic conditions such as hypertension and diabetes, where adjunctive digital interventions yielded superior outcomes [18-21]. Furthermore, our findings are consistent with previous observations that diseases heavily reliant on patient self-management exhibit greater differential treatment effects with adjunctive DTx use [12,22].

In terms of MMO, the DTx group exhibited a larger improvement, likely attributable to the structured exercise modules included in the app. This pattern corresponded with pain reduction and functional improvement, as indicated by the JFLS-20 scores, reflecting consistency among key clinical measures. Notably, pain relief was accompanied by improved functional capacity and subjective perception of functional limitations, demonstrating a coherent pattern across symptomatic and functional outcomes [23]. These results indicate that patients not only experienced pain relief but also subjectively recognized improvements in their condition. The alignment between objective clinical outcomes and patient perception reinforces the therapeutic value of DTx in enhancing both physical function and patient-reported experiences.

OBC outcomes indicated that parafunctional behaviors declined in both groups, with a greater mean reduction in the DTx group than in the sham group, although the between-group difference in change scores did not reach statistical significance. This finding suggests that DTx may effectively address modifiable behavioral risk factors, particularly through structured app-based components. In conventional care, strategies to guide or monitor behavioral changes between clinic visits are limited [8,9,24]. DTx facilitate continuous behavioral support through real-time tracking and feedback, enhancing motivation and leading to more consistent patient engagement [25]. This mechanism is analogous to the role of continuous glucose monitors in diabetes, which are known to improve behavioral adherence and outcomes. Such capabilities are typically not addressed in routine clinical care [26,27].

PHQ-4 scores decreased slightly in both groups, without significant between-group differences. Although the DTx app did not specifically target mental health, improvements in symptom burden may have contributed to secondary psychological benefits. While the effects on mental health were limited in terms of between-group differences, a statistically significant improvement was observed in the DTx group at week 6, suggesting the potential utility of DTx components in addressing psychological burden. Given the complex nature of psychological symptoms, longer and more targeted interventions may be necessary; however, our findings provide preliminary evidence of the feasibility of digital approaches in supporting mental health in TMD [28]. Additionally, for patients presenting with high psychological scores, DTx may offer a practical framework to support tailored interventions by providing structured, evidence-based approaches to mental health management. This principle also applies to behavioral outcomes such as those measured by the OBC, where individuals with high baseline scores may benefit from structured guidance and consistent behavioral feedback that conventional care often lacks [29].

Mechanistically, the DTx platform likely enhanced patient engagement through guided content, self-monitoring, and feedback. These features support self-regulation and may promote sustainable behavioral modification [29]. Prior studies have demonstrated that structured behavioral interventions in TMD, including habit reversal training and self-care education, can lead to meaningful clinical improvements [11,30]. The observed efficacy of the DTx platform in this trial is consistent with these findings, suggesting that digital formats can facilitate and scale such evidence-based strategies. Importantly, these benefits were achieved without additional clinical burden, highlighting the feasibility of DTx integration into routine care.

### Strengths and Limitations

Methodologically, to our knowledge, this study is among the first to apply a double-blind, sham-controlled design to a DTx trial for orofacial pain. The use of a structurally identical sham app enabled rigorous control of the placebo effect [11]. Additional strengths include centralized randomization, strong adherence, and low rates of missing data (5/102, 5% participants), with only 9 participants overall excluded from the primary outcome analysis (n=5, 56% lost to follow-up and n=4, 44% excluded due to predefined protocol noncompliance).

However, this study has several limitations. First, the relatively short follow-up period limits the ability to assess the durability and sustainability of treatment effects, particularly in the context of chronic conditions such as TMD. Second, although overall adherence was acceptable, individual variation in digital literacy, device access, and environmental factors may have influenced user engagement and the consistency of intervention delivery [31]. Third, outcomes were based on patient-reported measures. However, reliance on patient-reported measures aligns with the prevailing methodology in TMD research, where objective clinical end points remain limited. Pain intensity (measured by the VAS) and MMO are widely used metrics in global TMD trials [15]. Moreover, the DC/TMD Axis II protocol endorsed by the National Institutes of Health prioritizes self-report instruments for psychosocial and behavioral assessment, highlighting their accepted clinical relevance despite the subjectivity [17,32]. Fourth, with the exception of minor baseline differences in OBC and PHQ-4 scores, randomization achieved comparable distributions across groups, as no statistically significant differences were observed in other key variables, including VAS pain, MMO, and demographic characteristics. These minor imbalances were not clinically meaningful and are unlikely to have influenced the study outcomes. The observed differences in OBC and PHQ-4 scores were small relative to their full scale ranges and below established clinical cutoffs. Given the modest sample size, additional adjusted analyses, such as analysis of covariance or mixed models, would have been limited in statistical power. Participants were recruited from 2 institutions, and although group allocation was reasonably balanced, the sample size may still have permitted some random imbalances. Finally, although conventional treatment was maintained across study sites, heterogeneity in clinical practice patterns or patient interactions could not be entirely controlled. This reflects a common challenge in real-world clinical trials and underscores the need to balance ecological validity with protocol standardization [33]. Nevertheless, such pragmatic variability is inevitable in studies intended for widespread clinical application. For a DTx model to be broadly applicable in TMD care, analyses such as those performed in this study are essential at the initial stage. Further research should validate these findings in more narrowly defined patient subgroups and clinical scenarios to refine and tailor DTx implementation.

### Future Directions

Clinically, DTx represent a practical and scalable adjunct to conventional care, especially for managing behavioral and psychosocial components of TMD that are often underaddressed in routine clinical settings. The ability of DTx to deliver structured interventions remotely can help overcome limitations in provider availability and patient access, while also promoting consistent engagement outside of clinic visits [34]. These features are particularly relevant in chronic conditions such as TMD, where continuous self-management plays a critical role [35].

Future investigations should include extended follow-up periods to evaluate the long-term clinical outcomes and sustainability of DTx therapeutic effects. In addition, the incorporation of AI-driven analytics into DTx platforms holds promise for optimizing patient engagement through real-time feedback, symptom-based personalization, and adaptive content delivery. Such enhancements may improve both adherence and clinical efficacy across heterogeneous patient populations.

## Appendix

Multimedia Appendix 1 Characteristics of participants included in the per-protocol analysis.

Multimedia Appendix 2 Higher resolution version of Figure 3. Primary and secondary outcomes. JFLS-20: Jaw Functional Limitation Scale-20; MMO: maximum mouth opening; OBC: Oral Behavior Checklist; PHQ-4: Patient Health Questionnaire-4; VAS: visual analog scale.

Multimedia Appendix 3 Detailed results of the intention-to-treat analysis.

Multimedia Appendix 4 CONSORT-eHEALTH checklist (V 1.6.1).

## Abbreviations

CONSORT-EHEALTH: Consolidated Standards of Reporting Trials of Electronic and Mobile Health Applications and Online Telehealth
DC/TMD: diagnostic criteria for temporomandibular disorders
DTx: digital therapeutic
ITT: intention-to-treat
JFLS-20: Jaw Functional Limitation Scale-20
MMO: maximum mouth opening
OBC: Oral Behavior Checklist
PHQ-4: Patient Health Questionnaire-4
TAU: treatment as usual
TMD: temporomandibular disorder
VAS: visual analog scale

## Data Availability

The datasets generated or analyzed during this study are available from the corresponding author on reasonable request.
