# Supplementary material for: Evaluating the Efficacy of a Digital Therapeutic Intervention for Temporomandibular Disorders: Multicenter, Randomized, Sham-Controlled Trial
Source: J Med Internet Res. 2025 Oct 24;27:e83545. doi: 10.2196/83545 (PMC12595392; doi:10.2196/83545)
Supplement: Multimedia Appendix 1 [file jmir_v27i1e83545_app1.docx]

**Table**. Demographic Characteristics (PP analysis)

|  | **DTx group  N=44** | | **Sham control group  N=49** | | ***P*-value** |
| --- | --- | --- | --- | --- | --- |
| **Age (yr)** |  |  |  |  |  |
| Mean (SD) | 36.89(11.65) |  | 40.67(12.41) |  | 0.13^†^ |
| Min, Max(Range) | 35.5(19, 65) |  | 40(19, 64) |  |  |
| **Sex** | N | % | N | % |  |
| Male | 13 | 26.00 | 17 | 32.69 | 0.59^††^ |
| Female | 31 | 62.00 | 32 | 61.54 |  |
| **Height (cm)** |  |  |  |  |  |
| Mean (SD) | 164.80(8.41) |  | 166.67(9.22) |  | 0.31^†^ |
| Min, Max(Range) | 163(150, 179) |  | 165(150, 188) |  |  |
| **Weight (kg)** |  |  |  |  |  |
| Mean (SD) | 62.88(12.42) |  | 66.92(15.67) |  | 0.17^†^ |
| Min, Max(Range) | 60(44, 95) |  | 64(45, 110) |  |  |
| **Smoking status** | N | % | N | % |  |
| Never smoker | 37 | 74.00 | 41 | 78.85 | 0.75^††^ |
| Former smoker | 4 | 8.00 | 6 | 11.54 |  |
| Current smoker | 3 | 6.00 | 2 | 3.85 |  |
| **Cigarette consumption (per week)** |  |  |  |  |  |
| Former smoker |  |  |  |  |  |
| Mean (SD) | 28.00(29.47) |  | 65.33(42.74) |  | 0.17^†^ |
| Min, Max(Range) | 20.5(1, 70) |  | 65(12, 140) |  |  |
| Current smoker |  |  |  |  |  |
| Mean (SD) | 45.00(40.93) |  | 57.50(60.10) |  | 0.79^†^ |
| Min, Max(Range) | 35(10, 90) |  | 57.5(15, 100) |  |  |
| **Alcohol consumption status** | N | % | N | % |  |
| Never drinker | 14 | 28.00 | 18 | 34.62 | 0.5^††^ |
| Former drinker | 6 | 12.00 | 3 | 5.77 |  |
| Current drinker | 24 | 48.00 | 28 | 53.85 |  |
| **Alcohol intake (per month)** |  |  |  |  |  |
| Former drinker |  |  |  |  |  |
| Mean (SD) | 6.50(7.04) |  | 18.67(12.22) |  | 0.09^†^ |
| Min, Max(Range) | 4(1, 20) |  | 16(8, 32) |  |  |
| Current drinker |  |  |  |  |  |
| Mean (SD) | 10.71(13.75) |  | 7.29(7.93) |  | 0.27^†^ |
| Min, Max(Range) | 4(1, 48) |  | 4(1, 32) |  |  |

† Statistical significance test for differences between groups at screening using independent two-sample t-test.

†† Statistical significance test for differences between groups at screening was performed using the chi-square test or Fisher’s exact test, as appropriate.

*Indicates statistical significance at *P*<.05
