# Supplementary material for: Evaluating the Efficacy of a Digital Therapeutic Intervention for Temporomandibular Disorders: Multicenter, Randomized, Sham-Controlled Trial
Source: J Med Internet Res. 2025 Oct 24;27:e83545. doi: 10.2196/83545 (PMC12595392; doi:10.2196/83545)
Supplement: Multimedia Appendix 3 [file jmir_v27i1e83545_app3.docx]

**Supplementary Table : ITT Analysis with LOCF Imputation**

**Table 1**. Comparison of outcomes between DTx and Sham groups over time

|  | **DTx**  **Mean ± SD** | **Sham**  **Mean ± SD** | **DTx – Sham**  **(95% CI)** | **p-value** |
| --- | --- | --- | --- | --- |
| **VAS** |  |  |  |  |
| Baseline | 50.70 ± 21.92 | 48.33 ± 18.35 | 2.37 (-5.61, 10.34) | 0.373^a^ |
| Week 2 | 36.10 ± 21.36 | 42.69 ± 21.68 | -6.59 (-15.09, 1.91) | 0.150^a^ |
| Week 4 | 29.60 ± 20.07 | 43.35 ± 24.43 | -13.85 (-22.69, -5.01) | 0.005^a*^ |
| Week 6 | 19.40 ± 19.24 | 38.47 ± 25.41 | -21.44 (-31.28, -11.59) | <0.001^a*^ |
| **MMO** |  |  |  |  |
| Baseline | 40.30 ± 6.89 | 39.25 ± 7.91 | 1.05 (-1.88, 3.99) | 0.477 |
| Week 2 | 43.00 ± 7.55 | 40.94 ± 8.12 | 2.08 (-1.04, 5.15) | 0.190 |
| Week 4 | 44.32 ± 7.26 | 40.93 ± 8.56 | 3.39 (0.22, 6.56) | 0.019^*^ |
| Week 6 | 45.85 ± 7.37 | 41.09 ± 8.44 | 4.73 (1.53, 7.93) | 0.004^*^ |
| **JFLS-20** |  |  |  |  |
| Baseline | 3.58 ± 2.03 | 3.63 ± 1.80 | 0.05 (-0.80, 0.71) | 0.778^a^ |
| Week 2 | 2.41 ± 1.68 | 3.17 ± 1.69 | -0.76 (-1.43, -0.10) | 0.030^a*^ |
| Week 4 | 1.90 ± 1.70 | 2.59 ± 1.79 | -0.69 (-1.39, 0.01) | 0.029^a*^ |
| Week 6 | 1.42 ± 1.57 | 2.36 ± 1.94 | -0.94 (-1.65, -0.23) | 0.008^a*^ |
| **OBC** |  |  |  |  |
| Baseline | 26.12 ± 8.81 | 21.63 ± 9.45 | 4.49 (0.88, 8.10) | 0.015^*^ |
| Week 1 | 25.34 ± 9.38 | 21.14 ± 9.10 | 4.2 (0.56, 7.85) | 0.024^*^ |
| Week 2 | 23.04 ± 9.82 | 20.51 ± 10.46 | 2.53 (-1.48, 6.54) | 0.213 |
| Week 3 | 22.51 ± 10.13 | 21.31 ± 11.06 | 1.20 (-3.02, 5.41) | 0.574 |
| Week 4 | 20.22 ± 10.26 | 20.55 ± 11.16 | 0.33 (-4.67, 4.02) | 0.980^a^ |
| Week 5 | 19.31 ± 11.13 | 20.88 ± 11.23 | -1.57 (-6.05, 2.91) | 0.555^a^ |
| Week 6 | 18.08 ± 11.84 | 20.12 ± 11.41 | -2.04 (-6.73, 2.65) | 0.274^a^ |
| **PHQ-4** |  |  |  |  |
| Baseline | 3.54 ± 2.25 | 2.80 ± 1.97 | 0.74 (-0.10, 1.57) | 0.089 |
| Week 1 | 3.52 ± 2.44 | 2.63 ± 2.20 | 0.89 (-0.02, 1.81) | 0.064^a^ |
| Week 2 | 3.22 ± 2.47 | 2.47 ± 1.88 | 0.75 (-0.12, 1.61) | 0.144^a^ |
| Week 3 | 3.41 ± 2.65 | 2.41 ± 2.37 | -0.01 (-0.70, 0.068) | 0.028^a*^ |
| Week 4 | 3.10 ± 2.43 | 2.63 ± 2.46 | 0.47 (-0.51, 1.45) | 0.206^a^ |
| Week 5 | 3.06 ± 3.03 | 2.65 ± 2.67 | 0.41 (-0.74, 1.55) | 0.534^a^ |
| Week 6 | 2.98 ± 2.51 | 2.37 ± 2.43 | 0.61 (-0.38, 1.61) | 0.147^†^ |

^a^ p-value was calculated using the Wilcoxon rank-sum test due to non-normal distribution; all other p-values were obtained using the independent two-sample t-test.

*Indicates statistical significance at p < 0.05.

SD, standard deviation; CI, confidence interval; VAS, visual analog scale; JFLS-20, jaw functional limitation scale; MMO, maximum mouth opening; OBC, Oral Behavior Checklist; PHQ-4, Patient Health Questionnaire-4

**Table 2**. Comparison of changes from baseline between DTx and Sham groups over time

|  | **ΔDTx** | | **ΔSham** | | **ΔDTx - ΔSham**  **(95% CI)** | **p-value** |
| --- | --- | --- | --- | --- | --- | --- |
|  | **Mean ± SD** | **p-value^a^** | **Mean ± SD** | **p-value^a^** |  |  |
| **VAS** |  |  |  |  |  |  |
| Week 2 | -14.60 ± 22.61 | <0.001^*^ | -5.65 ± 19.30 | 0.080 | -8.95 (-17.25, -0.66) | 0.003^b*^ |
| Week 4 | -21.10 ± 22.91 | <0.001^*^ | -4.88 ± 22.75 | 0.163 | -13.85 (-22.69, -5.01) | 0.006^b*^ |
| Week 6 | -33.64 ± 25.34 | <0.001^*^ | -9.86 ± 24.96 | 0.008^*^ | -23.78 (-34.15, -13.41) | <0.001^*^ |
| **MMO** |  |  |  |  |  |  |
| Week 2 | 2.70 ± 5.68 | 0.002^*^ | 1.70 ± 5.54 | 0.037^*^ | 1.00 (-1.21, 3.22) | 0.248^b^ |
| Week 4 | 4.21 ± 5.89 | <0.001^*^ | 1.50 ± 6.13 | 0.090 | 2.71 (0.32, 5.11) | 0.019^b*^ |
| Week 6 | 5.66 ± 5.81 | <0.001^*^ | 1.57 ± 6.20 | 0.082 | 4.08 (1.66, 6.51) | <0.001^b*^ |
| **JFLS-20** |  |  |  |  |  |  |
| Week 2 | -1.18 ± 1.68 | <0.001^*^ | -0.46 ± 1.42 | 0.023^*^ | -0.71 (-1.33, -0.10) | 0.029^b*^ |
| Week 4 | -1.75 ± 1.91 | <0.001^*^ | -1.00 ± 1.57 | <0.001^*^ | -0.74 (-1.44, -0.05) | 0.036^b*^ |
| Week 6 | -2.20 ± 1.92 | <0.001^*^ | -1.26 ± 1.88 | 0.001^*^ | -0.94 (-1.71, -0.17) | 0.017^b*^ |
| **OBC** |  |  |  |  |  |  |
| Week 1 | -0.78 ± 7.53 | 0.467 | -0.49 ± 5.11 | 0.496 | 0.29 (-2.25, 2.83) | 0.821 |
| Week 2 | -3.08 ± 7.94 | 0.008^*^ | -1.12 ± 6.92 | 0.254 | -1.96 (-4.90, 0.98) | 0.188 |
| Week 3 | -3.74 ± 9.16 | 0.006^*^ | -0.31 ± 7.89 | 0.778 | -3.42 (-6.81, 0.03) | 0.047^*^ |
| Week 4 | -6.02 ± 10.26 | <0.001^*^ | -1.57 ± 8.01 | 0.176 | -4.45 (-8.14, -0.76) | 0.019^*^ |
| Week 5 | -6.94 ± 11.20 | <0.001^*^ | -1.25 ± 8.14 | 0.290 | -5.69 (-9.62, -1.77) | 0.005^*^ |
| Week 6 | -8.17 ± 12.29 | <0.001^*^ | -2.00 ± 7.48 | 0.067 | -6.17 (-10.29, -2.04) | 0.004^*^ |
| **PHQ-4** |  |  |  |  |  |  |
| Week 1 | -0.02 ± 2.16 | 0.795 | -0.18 ± 1.68 | 0.440 | -0.16 (-0.92, 0.61) | 0.496^b^ |
| Week 2 | -0.32 ± 2.02 | 0.267 | -0.33 ± 1.42 | 0.118 | 0.01 (-0.68, 0.12) | 0.815^b^ |
| Week 3 | -0.14 ± 2.57 | 0.553 | -0.39 ± 2.02 | 0.114 | -0.25 (-1.16, 0.67) | 0.743^b^ |
| Week 4 | -0.45 ± 2.31 | 0.090 | -0.14 ± 2.11 | 0.502 | -0.31 (-1.19, 0.58) | 0.447^b^ |
| Week 5 | -0.49 ± 2.79 | 0154 | -0.12 ± 2.18 | 0.667 | -0.37 (-1.37, 0.64) | 0.316^b^ |
| Week 6 | -0.56 ± 2.12 | 0.047^*^ | -0.41 ± 2.15 | 0.203 | -0.15 (-1.02, 0.71) | 0.566^b^ |

^a^ Statistically significant changes from baseline to each week were tested using the paired t-test.

^b^ p-value was calculated using the Wilcoxon rank-sum test due to non-normal distribution; all other p-values were obtained using the independent two-sample t-test.

*Indicates statistical significance at p < 0.05.

SD, standard deviation; CI, confidence interval; VAS, visual analog scale; JFLS-20, jaw functional limitation scale; MMO, maximum mouth opening; OBC, Oral Behavior Checklist; PHQ-4, Patient Health Questionnaire-4
